# Application of the Crystalline Sponge Method to Revise the Structure of the Phenalenone Fuliginone

**DOI:** 10.3390/molecules22020211

**Published:** 2017-01-30

**Authors:** Robert Brkljača, Bernd Schneider, William Hidalgo, Felipe Otálvaro, Felipe Ospina, Shoukou Lee, Manabu Hoshino, Makoto Fujita, Sylvia Urban

**Affiliations:** 1 School of Science (Applied Chemistry and Environmental Science), RMIT University, GPO Box 2476, Melbourne, Victoria 3001, Australia; robert.brkljaca@rmit.edu.au; 2 Max-Planck-Institute for Chemical Ecology, Beutenberg Campus, Hans-Knöll-Str. 8, D-07745 Jena, Germany; schneider@ice.mpg.de (B.S.); williamhidalgob@hotmail.com (W.H.); 3 Instituto de Química, Síntesis y Biosíntesis de Metabolitos Naturales, Universidad de Antioquia, AA 1226 Medellín, Colombia; leon.otalvaro@udea.edu.co (F.Ot.); arafron@hotmail.com (F.Os.); 4 Department of Applied Chemistry, School of Engineering, The University of Tokyo, Hongo, Bunkyo-ku, Tokyo 113-8656, Japan; slee@g.ecc.u-tokyo.ac.jp (S.L.); mhoshino@appchem.t.u-tokyo.ac.jp (M.H.); mfujita@appchem.t.u-tokyo.ac.jp (M.F.)

**Keywords:** fuliginone, phenylphenalenone, synthesis, crystalline sponge method, HPLC-NMR

## Abstract

The structure of fuliginone was revised from a phenyl substituted phenalenone to a hydroxyl substituted phenalenone as a result of its re-purification via HPLC with subsequent NMR analysis together with an independent synthesis and analysis of the crystal structure, which was secured via the crystalline sponge method. On-flow High Performance Liquid Chromatography coupled to Nuclear Magnetic Resonance spectroscopy (HPLC-NMR) was employed to confirm the presence of the natural product in the plant extract and to monitor for any possible degradation or conversion of the compound.

## 1. Introduction

Phenylphenalenones are specialized natural products mainly occurring in the Haemodoraceae [1,2] and the Musaceae [3]. The involvement in the plant’s defense against phytopathogenic fungi [4,5] and nematodes [6] would appear to be the major ecological function of phenylphenalenones. Pharmacological [7], antimicrobial [1,8] and radical scavenging activities [9] have been also reported. Therefore, new phenylphenalenones and related compounds from natural [1,8,10] or synthetic sources [11,12] are desirable.

Fuliginone is a phenalenone-type compound that was recently reported from the Australian plant *Macropidia fuliginosa* [1]. At the time of reporting this compound, it was recognised that a synthetic compound with the same structure to that of fuliginone existed, but that there were some discrepancies in the NMR data of the two compounds [13]. To investigate this further a number of approaches were adopted, including unequivocally securing the structures of fuliginone and the synthetic compound via the crystalline sponge method, conducting additional NMR analysis on the synthetic phenylphenalenone and also for fuliginone after re-purification via HPLC.

The crystalline sponge method is a recently-developed X-ray technique that can analyze the structure of various molecules, by absorbing the guest into the ordered cavities of crystalline porous complexes (crystalline sponges) [14,15,16,17,18,19].

This technique has recently been applied to deduce the absolute structure determination of scarcely available oily natural products, where no other means except for the crystalline sponge method could directly address the absolute configuration of the compounds [20,21,22].

**Figure 1:**
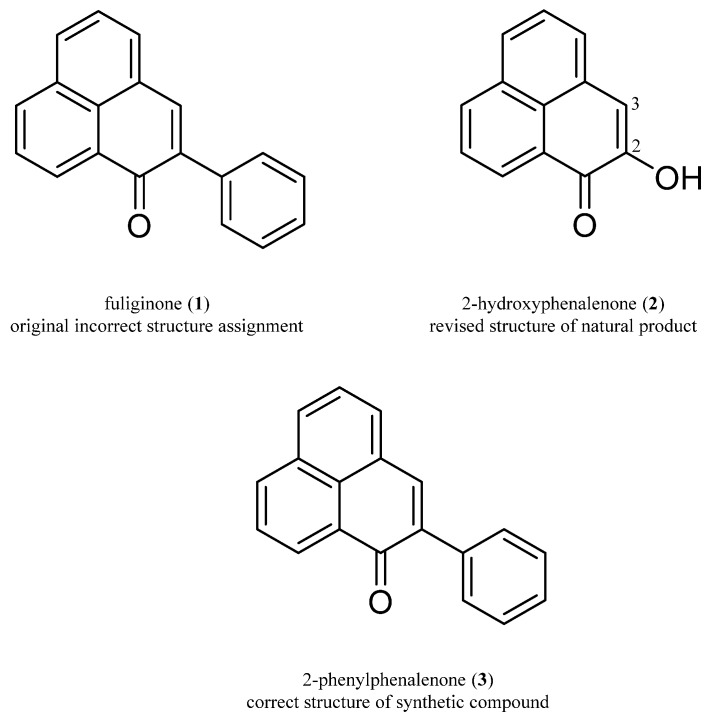
Structure of incorrect and revised structures.

In addition, HPLC-NMR was also conducted on an enriched fraction of *M. fuliginosa* to confirm the presence of either fuliginone or 2-hydroxyphenalenone (or both), and to establish if any degradation or possible conversion had taken place during or after isolation. From these combined approaches it could be concluded that the structure for the natural product previously attributed the name fuliginone should be revised from that of a phenyl substituted phenalenone to a hydroxyl substituted phenalenone (Figure 1).

## 2. Results and Discussion

### 2.1. Comparison of NMR and MS Data of Isolated Natural Product and Synthetic Product

A phenalenone-type compound was isolated from the bulbs of *M. fuliginosa* and the structure was reported as a phenylphenalenone and attributed the name fuliginone (**1**) (Figure 1) on the basis of the NMR and high resolution mass spectrometry data [1]. A literature review indicated that this compound had previously been reported as a synthetic derivative [13]. However, the UV and NMR of the isolated compound (fuliginone) and the synthetic compound were not in agreement, despite the same structure being proposed for both compounds. Discrepancies in the NMR data were noted between the two compounds. Of significance was that the isolated natural product (fuliginone) displayed a proton signal at δ_H_ 7.17 and carbon signals at δ_C_ 149.5 and 113.8, whereas the synthetic derivative showed only proton signals above δ_H_ 7.40 and no carbon signals below δ_C_ 126.7 [13]. The UV maxima for the isolated compound (fuliginone) was observed at λ_max_ 431 nm [1], whilst the synthetic derivative displayed a UV maxima at λ_max_ 403 nm [13].

The NMR data of both compounds was re-analysed. The natural product, which had been stored at −20 °C for over a year, was reconstituted into *d*_6_-acetone and placed into a NMR tube. NMR analysis showed that the proton spectrum was not identical to the spectrum obtained when the compound was originally isolated. The integration of the signals attributed to the terminal aromatic moiety, no longer supported this structure. Upon further inspection of the original NMR data of the isolated natural product [1], there were no gHMBCAD NMR correlations observed which supported the presence of a pendant aromatic moiety in fuliginone (**1**). This meant that it was necessary to re-purify the isolated natural product.

The isolated natural product was subjected to re-purification using reverse phased HPLC. Subsequent off-line NMR and MS analysis demonstrated that the isolated compound was in fact 2-hydroxyphenalenone (**2**) and not the phenylphenalenone structure originally assigned to fuliginone (**1**) (Figure 1). It is believed that there was a co-eluting compound present in the original isolation of the natural product which was responsible for the additional aromatic signals observed in the proton NMR spectrum that was originally assigned to position 2. This co-eluting compound also led to a higher molecular mass being observed for a pseudomolecular ion at *m/z* 257 [M + H]^+^.

The NMR data of the re-purified natural product matched those of 2-hydroxyphenalenone [5] (see Supporting Information). In particular the chemical shifts of H-3 (δ_H_ 7.17), C-2 (δ_C_ 149.5) and C-3 (δ_C_ 113.8) for the isolated natural product [1] were in accordance with those of 2-hydroxyphenalenone (δ_H-3_ 7.20, δ_C-2_ 150.0 and δ_C-3_ 114.2) [5]. The structure was further confirmed by HMBC cross signals of H-3 with C-2 and C-1 (δ_C_ 180.9) and ROESY correlations between H-3 and H-4 (δ_H_ 7.84). 2-Hydroxyphenalenone (**2**) is a natural product previously isolated and reported from *Musa acuminata* var. “Yangambi km 5’’ (AAA) (Musaceae)” [5] which is a family of plants related to that of *M. fuliginosa* (Haemodoraceae).

Since the structure of the re-purified natural product had been revised, 2-phenylphenalenone (**3**) was synthesized by a Suzuki-Miyaura coupling using 2-bromoperinaphthenone as the starting material. The chemical synthesis finally confirmed the structure determined by NMR and MS to be 2-phenylphenalenone (**3**). In particular the NMR data of this synthetic product were in agreement to that previously reported [13]. Comparison of the proton and carbon chemical shifts of 2-hydroxyphenalenone (**2**) and 2-phenylphenalenone (**3**) showed noticeable differences at positions 2 and 3 of the structures. Comparison of the NMR data of both compounds in CDCl_3_, indicated that the signals of H-3 occurred at δ_H_ 7.17 and 7.85 respectively for compounds (**2**) and (**3**). The carbon signals of C-2 and C-3 occurred at δ_C_ 149.5 and 139.4; and δ_C_ 113.8 and 139.5 respectively for compounds (**2**) and (**3**). It is clear that the differences in the carbon and proton chemical shifts between compounds (**2**) and (**3**) are significant and that the two compounds can be easily distinguished using these key NMR signals.

### 2.2. Crystalline Sponge Method

Concurrently, as the re-purified natural product and synthetic product were being compared via NMR and MS methods, the isolated natural product (fuliginone) as well as the synthetic derivative were also subjected to analysis using the crystalline sponge method, which is able to secure the single crystal structure of non-crystalline compounds [16,22].

**Figure 2:**
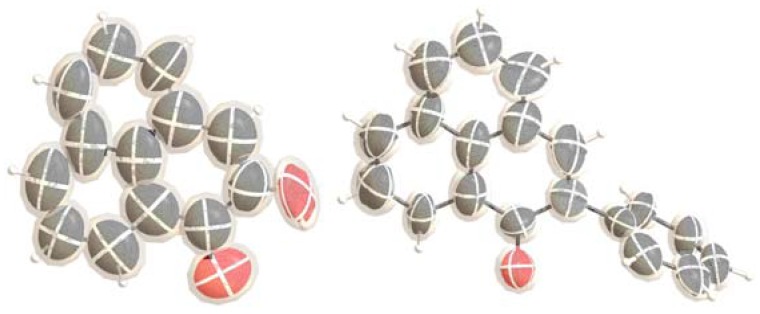
ORTEP diagram (50% probability) of isolated natural product (**left**) and synthetic product (**right**).

**Figure 3:**
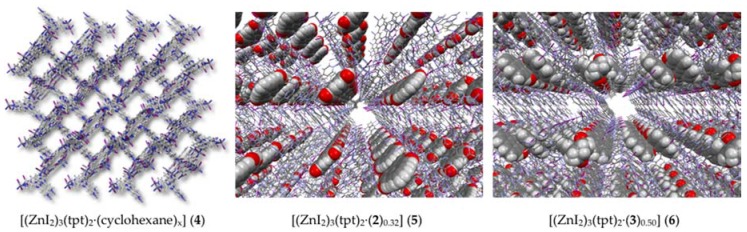
Crystal structure of the guest-absorbed crystalline sponge.

The isolated natural product was successfully absorbed into the crystalline sponge, and the structure was determined via single crystal X-ray diffraction. The results of the crystalline sponge method (Figure 2) indicated that the correct structure of the isolated natural product was not a 2-substituted phenylphenalenone (**1**) but rather the 2-hydroxyphenalenone (**2**). As the structure did not support the original identity of the natural product, the natural product was subjected to GC-MS analysis prior to conducting the crystalline sponge method study. One major and one minor component were observed with the major component displaying a *m/z* 196 [M]^+^. This, together with the single crystal X-ray data (Figure 3), was in agreement with the structure secured following reanalysis of the NMR and MS data after re-purification of the original isolated natural product.

The synthetic 2-phenylphenalenone (**3**) was also subjected to the crystalline sponge method. Subsequent single crystal X-ray diffraction of the synthetic compound confirmed its structure to be 2-phenylphenalenone (**3**) (Figure 1 and also see Supporting Information).

### 2.3. Chemical Profiling of Natural Products Present in the Bulbs of M. fuliginosa

When the natural product (formerly attributed the name fuliginone) was first isolated, the integration of the aromatic region supported the presence of five protons, suggesting the occurrence of an aromatic moiety. The natural product had also been stored at −20 °C for over 1 year. To ensure that fuliginone (**1**) had not degraded during storage, a fresh extraction of the bulbs of *M. fuliginosa* was carried out to determine if the natural product was in fact a 2-phenylphenalenone or 2-hydroxyphenalenone (**2**). An enriched silica column fraction of the dichloromethane extract was subjected to on-flow HPLC-NMR and HPLC-MS analysis. Each of the compounds present in the enriched fraction was identified and consequently, only 2-hydroxyphenalenone (**2**) was observed. This confirmed that the structure originally attributed to fuliginone (**1**) as 2-phenylphenalenone is not a natural product that occurs in this plant and that co-eluting material was responsible for the original incorrect structure proposed. 

## 3. Materials and Methods

### 3.1. General Experimental Procedures

All organic solvents used were analytical reagent (AR or GR), UV spectroscopic, or HPLC grades with Milli-Q water also being used. Offline ^1^H (500 MHz), and ^13^C (125 MHz), COSY, HSQC, HMBC and 2D ROESY spectra were acquired in *d*_6_-acetone at 300 K on a Bruker AVANCE III HD 500 NMR spectrometer (Bruker-Biospin, Karlsruhe, Germany) equipped with a TCI 5 mm cryoprobes. The residual solvent signals were used for referencing spectra in the ^1^H and ^13^C dimensions. NMR data were processed with Bruker Topspin software, version 3.2. Silica gel flash chromatography was carried out using Davisil LC35Å silica gel (40–60 mesh) with a 20% stepwise solvent elution from 100% petroleum spirits (60–80 °C) to 100% CH_2_Cl_2_ to 100% EtOAc and finally to 100% MeOH. HRESIMS was recorded with a LTQ-Orbitrap XL mass spectrometer (Thermo Fisher Scientific, Bremen, Germany). Electrospray ionization (ESI) source parameters were set to 4 kV for spray voltage, 35 V for transfer capillary voltage at a capillary temperature of 275 °C. The samples were measured in positive ion mode in the mass range of *m/z* 100 to 1500 using 30,000 m/Δm resolving power.

### 3.2. Plant Material

The plant material was of the same batch as that referred to in Brkljača, White and Urban (2015) [1].

### 3.3. Natural Product Material

The natural product originally isolated and attributed the name fuliginone was obtained from the previous study of *M. fuliginosa*. The details of the isolation can be found in Brkljača, White and Urban (2015) [1].

### 3.4. Re-purification of Natural Product Material

The impure natural product material was re-purified to yield 2-hydroxyphenalenone (**2**). Semi-preparative HPLC was carried out on an Agilent 1100 chromatography system (quaternary solvent delivery pump G1311A, autosampler G1313A (Agilent Technologies, Waldbronn, Germany) and a photodiode array detector (200–700 nm) (J&M Analytik AG, Aalen, Germany). 

The chromatography system was equipped with a LiChrospher 100 C_18_ HPLC column (LiChroCART, 250 mm × 4 mm, 5 µm). The solvent gradient used was 0 min: 30% MeCN (0.1% TFA), 50 min: 90% MeCN (0.1% TFA), 55 min: 30% MeCN (0.1% TFA) with a flow rate of 1 mL·min^−1^. The detection wavelength was 254 nm.

### 3.5. Crystalline Sponge Method

The crystalline sponge [(ZnI_2_)_3_(tpt)_2_·(cyclohexane)_x_] (tpt = 1,3,5-tris(4-pyridyl)triazine) (**4**) was prepared according to the reported procedure [23]. A screw-top microvial (Osaka chemical, cat. No. 11090620), screw cap with septum seal (Osaka chemical, cat. No. 53951-09FB) and a syringe needle (TERUMO, cat. No. NN-2116R) were used for guest inclusion into the porous crystals.

A single crystal of porous complex [(ZnI_2_)_3_(tpt)_2_·(cyclohexane)_x_] (**4**), immersed in cyclohexane (45 μL) in a small vial, was treated with 1,2-dichloroethane solution (5 μL) of **2** (1 μg·μL^−1^, 5 μg) for 1 day at 50 °C for the uptake of **2** into the pores of **4**. The thus prepared red single crystal of **2**·**4** complex (**5**) with a dimension of 325 × 135 × 88 μm^3^ was used for the X-ray diffraction data collection. A single crystal X-ray diffractometer (SuperNova by Rigaku Oxford Diffraction, Tokyo, Japan) equipped with a fine-focused Cu *Kα* X-ray source (Nova, Tokyo, Japan) and a high-sensitivity CCD detector (Altas S2 CCD detector, Tokyo, Japan) was used for the diffraction data collection of **5**.

Crystallographic data of **5**. C_58.11_H_62.53_O_0.63_N_12_I_6_Zn_3_, M = 1896.70, monoclinic *C*2/*c*, a = 34.4155(14) Å, b = 14.8927(4) Å, c = 30.6402(11) Å, β = 100.849(4)°, V = 15423.6(10) Å^3^, Z = 8, GoF = 1.041, *R*_1_ = 0.0685, *wR*_2_ = 0.2250. CCDC deposit number 1479613. The CCDC data can be obtained free of charge from the Cambridge Crystallographic Data Centre (http://www.ccdc.cam.ac.uk/data_request/cif, Cambridge, United Kingdom).

A single crystal of porous complex **4**, immersed in cyclohexane (45 μL) in a small vial, was treated with 1,2-dichloroethane solution (1 μL) of **3** (1 μg·μL^−1^, 1 μg) for 1 day at 50 °C then 2 days at room temperature for the uptake of **3** into the pores of **4**. The thus prepared single crystal of **3**·**4** complex (**6**) with a dimension of 200 × 120 × 80 μm^3^ was used for the X-ray diffraction data collection. A single crystal X-ray diffractometer (Rigaku XtaLAB P200, Tokyo, Japan) equipped with a rotating anode X-ray source (Mo *K*α radiation, Rigaku, Tokyo, Japan) and a hybrid photon counting detector (DECTRIS Pilatus 200K, Tokyo, Japan) was used for the diffraction data collection of **6**.

Crystallographic data of **6**. C_65_H_69_N_12_I_6_Zn_3_, M = 1983.83, monoclinic *C*2/*c*, a = 34.6020(9) Å, b = 15.0086(3) Å, c = 30.0555(6) Å, β = 101.483(2)°, V = 15296.2(6) Å^3^, Z = 8, GoF = 1.098, *R*_1_ = 0.0554, *wR*_2_ = 0.1506. CCDC deposit number 1479614. The CCDC data can be obtained free of charge from the Cambridge Crystallographic Data Centre (http://www.ccdc.cam.ac.uk/data_request/cif, Cambridge, United Kingdom).

### 3.6. GC/MS Identification of 2-Hydroxyphenalen-1-one

GC-MS analyses were performed with a GCMS-QP2010 Plus (SHIMADZU, Kyoto, Japan) spectrometer coupled with a GCMS-QP2010 Ultra (SHIMADZU, Kyoto, Japan). The autosampler was an AOC-20i (SHIMADZU, Kyoto, Japan). The injector was held at 250 °C and the injection volume was 1 µL (0.6 mg·mL^−1^ in dichloromethane) (splitless time 1.0 min). The instrument was equipped with an Inert Cap Pure-WAX capilary column of 60 m length, 0.32mm i.d. and 0.50 µm film thickness. The carrier gas was helium (purity 99.995%) at a constant pressure rate of 200kPa. The GC oven program started at 70 °C (1.0 min hold) ramped up to 250 °C (heating rate 10 °C·min^−1^) (hold time 71 min). The ion source temperature was 200 °C and the interface temperature was 250 °C. The ion trap scanned from 5.0 min to 90.0 min at a mass range from *m*/*z* 45.0 to 300 (EI, 1.25 kV). From 0 min to 5.0 min the MS was switched off (solvent delay). Data analysis and instrument control were carried out with GCMS solution 4.20 (SHIMADZU, Kyoto, Japan) software.

### 3.7. Identification of 2-Hydroxyphenalen-1-one in the Bulbs of M. fuliginosa

The bulbs of *M. fuliginosa* were extracted with 3:1 MeOH/CH_2_Cl_2_ (1 L). The crude extract was decanted and concentrated under reduced pressure and sequentially solvent partitioned (triturated) into CH_2_Cl_2_ and MeOH soluble extracts respectively. The CH_2_Cl_2_ extract was subjected to flash silica gel column chromatography (20% stepwise elution from petroleum ether (60–80 °C) to CH_2_Cl_2_ to EtOAc and, finally, to MeOH). The second 80% CH_2_Cl_2_/EtOAc fraction yielded a mixture of compounds. This enriched dichloromethane fraction was subjected to on-flow HPLC-NMR and HPLC-MS. HPLC-NMR was carried out on a 500 MHz Agilent DD2 NMR spectrometer equipped with a Varian ^1^H[^13^C] pulsed field gradient flow probe with a 60 μL active volume flow cell coupled to a Varian Prostar 210 solvent delivery system, a Prostar 430 Autosampler, and a Prostar 335 PDA detector. The HPLC-NMR analyses were carried out using CORBA communication and operated with VnmrJ software. The 2H resonance observed from the D_2_O was used to obtain a field-frequency lock. The resonances from the HOD and the methyl of the acetonitrile were suppressed using the water enhanced through transverse gradients (WET) solvent suppression experiment [24]. The residual HOD resonance of D_2_O was referenced to 4.64 ppm. For on-flow HPLC-NMR, 50-μL injections (4986 µg) of the enriched fraction were injected onto an Agilent Eclipse Plus C_18_ (150 mm × 4.6 mm, 5 µm) column using a solvent composition of 50% CH_3_CN/D_2_O at a flow rate of 0.8 mL·min^−1^. HRESILCMS was carried out on an Agilent 6540 Series TOF system (ESI operation conditions of 8 L·min^−1^ N_2_, 325 °C drying gas temperature, and 3500 V capillary voltage) equipped with an Agilent 1200 Series LC solvent delivery module (50% CH_3_CN/D_2_O at a flow rate of 0.8 mL·min^−1^) in either the positive or negative ionization modes, using an Agilent Eclipse Plus C_18_ (150 mm × 4.6 mm, 5 µm) column. The instrument was calibrated using the “Agilent Tuning Mix” with purine as the reference compound and the Hewlett–Packard standard HP0921.

### 3.8. Synthesis of 2-Phenylphenalen-1-one and 2-Hydroxyphenalen-1-one

2-Hydroxyphenalen-1-one was synthesized by oxidation of perinaphthenone with *tert*-butylhydroperoxide as previously reported [5]. To a solution of perinaphthenone in benzene were added two portions of aqueous tert-butylhydroperoxide (70 wt%) and TritonB (40 wt%) in methanol. The resulting epoxide was converted to 2-hydroxyphenalen-1-one by means of *p*-toluenesulfonic acid monohydrate and the product purified by open column chromatography. 2-Phenylphenalen-1-one was obtained from 2-bromoperinaphthenone using a Suzuki-Miyaura coupling mediated by [1,3-bis(2,6-diisopropylphenyl)imidazole-2-ylidene](3-chloropyridyl)palladium(II)dichloride under microwave conditions in 98% yield [25].

### 3.9. On-Line (HPLC-NMR & HPLC-MS) Characterization of Compounds

2-Hydroxyphenalen-1one (**2**): R_t_ = 5.55 min; HPLC-NMR WET1D NMR (500 MHz, 50% CH_3_CN/D_2_O) obtained from stop-flow HPLC-NMR mode δ 9.05 (1H, d, *J* = 6.5 Hz, H-4*^a^), 8.81 (1H, d, *J* = 8.5 Hz, H-9*^b^), 8.46 (1H, d, *J* = 8.0 Hz, H-6*^a^), 8.28 (1H, dd, *J* = 7.0, 8.5 Hz, H-8^#b^), 8.23 (1H, d, *J* = 7.0 Hz, H-7*^b^), 8.06 (1H, dd, *J* = 6.5, 8.0 Hz, H-5^#a^), 7.66 (1H, s, H-3), *^#^ signals interchangeable, ^ab^ indicates signals are part of the same spin system; HPLC-MS *m/z* 195.0450, 197.0597 (calcd for C_13_H_7_O_2_, 195.0452; calcd for C_13_H_9_O_2_, 197.0597).

### 3.10. Off-Line Characterization of Compounds

*2-Hydroxyphenalen-1-one* (**2**); ^1^H-NMR (500 MHz, (CD_3_)_2_O) δ 8.64 (1H, d, *J* = 7.5 Hz, H-9), 8.43 (1H, d, *J* = 8.0 Hz, H-7), 8.06 (1H, d, *J* = 8.2 Hz, H-6), 7.89 (1H, dd, *J* = 8.0, 7.5 Hz, H-8), 7.84 (1H, d, *J* = 7.0 Hz, H-4), 7.66 (1H, dd, *J* = 8.2, 7.0 Hz, H-5), 7.20 (1H, s, H-3). ^13^C-NMR (125 MHz, (CD_3_)_2_O) δ 180.9 (C, C-1), 150.0 (C, C-2), 136.9 (CH, C-7), 132.4 (C, C-6a), 131.5 (CH, C-9), 130.8 (CH, C-4), 130.2 (CH, C-6), 128.9 (C, C-3a),127.9 (C, C-9a), 127.6 (CH, C-5), 127.2 (CH, C-8), 124.9 (C, C-9b), 114.2 (CH, C-3). HRESIMS 197.0601 (calcd for C_13_H_9_O_2_, 197.0597).

*2-Phenylphenalen-1-one* (**3**); ^1^H-NMR (500 MHz, (CD_3_)_2_O) δ 8.61 (1H, dd, *J* = 7.3, 1.2 Hz, H-9), 8.40 (1H, dd, *J* = 8.1, 1.0 Hz, H-7), 8.20 (1H, dd, *J* = 8.3, 1.0 Hz, H-6), 8.06 (1H, s, H-3), 8.03 (1H, d, *J* = 7.0 Hz, H-4), 7.90 (1H, dd, *J* = 8.1, 7.3 Hz, H-8), 7.73 (1H, dd, overlap, H-5), 7.72 (2H, m, H-2’/H-6’), 7.45 (2H, m, H-3’/H-5’), 7.39 (1H, m, H-4’); ^1^H-NMR (500 MHz, CDCl_3_) δ 8.71 (1H, dd, *J* = 7.3, 1.1 Hz, H-9), 8.21 (1H, d, *J* = 8.1, 1.1 Hz, H-7), 8.02 (1H, d, *J* = 8.1 Hz, H-6), 7.85 (1H, s, H-3), 7.80 (1H, dd, *J* = 8.1, 7.3 Hz, H-8), 7.79 (1H, d, *J* = 7.9 Hz, H-4), 7.68 (2H, m, H-2’/H-6’), 7.61 (1H, dd, *J* = 8.1, 7.9 Hz, H-5), 7.46 (2H, m, H-3’/H-5’), 7.40 (1H, m, H-4’); ^13^C-NMR (125 MHz, (CD_3_)_2_O) δ 183.9 (C, C-1), 140.5 (CH, C-3), 139.7 (C, C-2), 137.8 (C, C-1’), 135.8 (CH, C-7), 133.2 (C, C-6a), 132.8 (CH, C-4), 132.4 (CH, C-6), 131.2 (CH, C-9), 130.7 (C, C-9a), 130.1 (CH, C-2’/C-6’), 129.0 (C, C-3a), 128.9 (CH, C-3’/C-5’), 128.8 (CH, C-4’), 128.3 (CH, C-8), 128.1 (CH, C-5), 127.9 (C, C-9b); HRESIMS *m/z* 256.0888 (calcd for C_19_H_13_O, 257.0966).

## 4. Conclusions

The structure of fuliginone was revised via a concerted approach. Re-purification of the natural product followed by NMR, mass spectrometric analyses and single crystal X-ray structures secured via the crystalline sponge method permitted for the unequivocal re-assignment of the natural product as a 2-hydroxyphenalenone, which was finally proven by chemical synthesis. The crystalline sponge method provides a reliable method to resolve structure issues for natural products that are not normally amenable to conventional X-ray methodologies such as fuliginone.

## Supplementary Materials

The following are available online at http://www.mdpi.com/1420-3049/22/2/211/s1.
