# Supplementary material for: Application of the Crystalline Sponge Method to Revise the Structure of the Phenalenone Fuliginone
Source: Molecules. 2017 Jan 30;22(2):211. doi: 10.3390/molecules22020211 (PMC6155586; doi:10.3390/molecules22020211)
Supplement: Supplementary file 1 [file molecules-22-00211-s001.pdf]

Supplementary Materials: Application of the Crystalline Sponge Method to Revise the Structure of the Phenalenone Fuliginone

Robert Brkljača, Bernd Schneider, William Hidalgo, Felipe Otálvaro, Felipe Ospina, Shoukou Lee, Manabu Hoshino, Makoto Fujita and Sylvia Urban

1. X-ray Diffraction Analysis of Complex 5

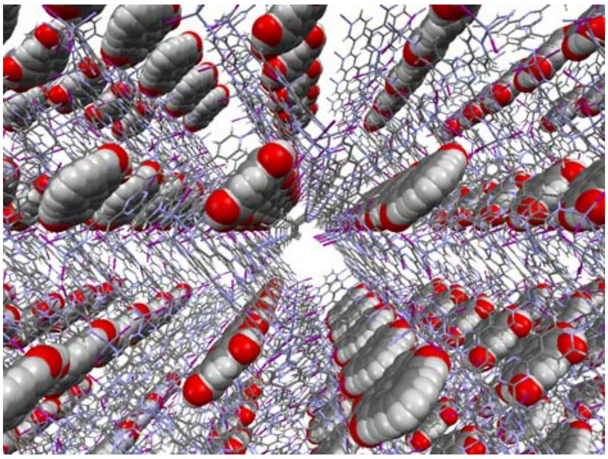

A single crystal X-ray diffractometer (SuperNova by Rigaku Oxford Diffraction) equipped with a fine-focused Cu *Kα* X-ray source (Nova) and a high-sensitivity CCD detector (Altas S2 CCD detector) was used for the diffraction data collection of **5**. The sample crystal was cooled to 100 K using a cold nitrogen stream (Cobra by Oxford Cryosystems). Two different detector positions (2θ = −41.46° or 112.00°) were applied for separately collecting diffraction data at the high-angle and low-angle regions. Exposure time for each diffraction image par 1° oscillation in the ω scan was selected to fulfil the suitable I/σ value; 116.13 sec to fulfil $I/\sigma > 5.1$ for the high-angle region and 21.57 sec to fulfil $I/\sigma > 14.92$ for the low-angle region. The sample-to-detector distance was 53 mm. On this setting, the maximum data resolution (*d*) is ca. 0.80 Å. The complete data collection took 24 h. Collected data were processed using the program CrysAlisPro ver. 1.171.38.41. In this process, numerical absorption correction based on Gaussian integration over a multifaceted crystal model was applied to correct significant Cu *Kα* X-ray absorption by the sample crystal. Empirical absorption correction using spherical harmonics was also applied. All crystal structures were solved using the program SHELXT ver. 2014/5 [1] and refined using the program SHELXL ver. 2014/7.[2] All non-hydrogen atoms were refined anisotropically. All hydrogen atoms are generated using the proper HFIX command and refined isotropically using the riding model. DFIX and DANG commands were applied to a guest compound based on the chemical information from NMR and mass-spectrometry data (Figure S1). Solvent cyclohexane molecules suggested in the electron density map were refined using some restraints (DFIX, DANG, SIMU, and ISOR) because corresponding electron densities were obscured due to their thermal motion and disordering. Short intermolecular interactions were found by the HTAB command.

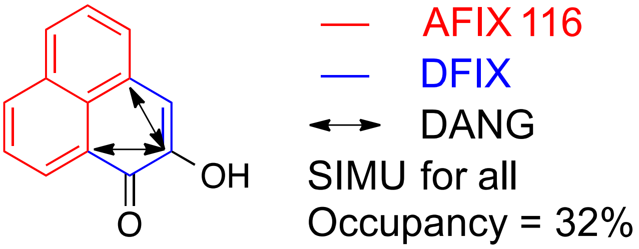

Figure S1. Restrain/constrain chart of compound **2**.

Under the refinement of X-ray diffraction data, overlapped cyclohexane molecule over the guest compound was expected as 18% population in an asymmetric unit however we could not find suitable remaining electron densities consisting cyclohexane molecule. Thus we tried to refine the population of the guest compound as 50% but the structure of **2** was reconstructed as chemically irrelevant one. Therefore the population of the guest compound should be 32% and disordered cyclohexanes, which occupancy was very low, should be overlapped over the guest compound. Planar structure consisting of remaining electron densities was still observed (Figure S2, red square). Although it was considered as another guest compound we could not construct it as a 2-hydroxy-1*H*-phenalen-1-one. This would be mainly because its occupancy should be extremely low.

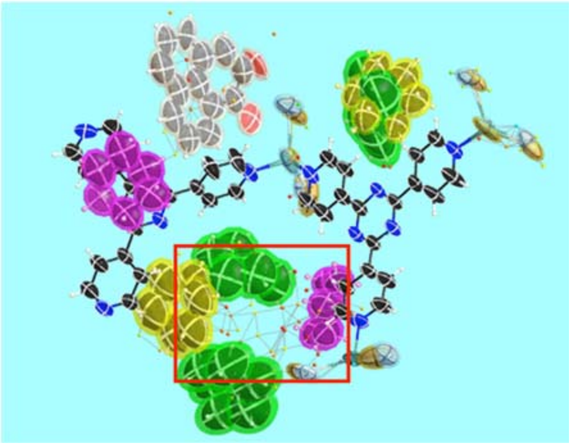

Figure S2. ORTEP diagram (50% probability) with remaining 100 Q-peaks.

2. X-ray Diffraction Analysis of Complex 6

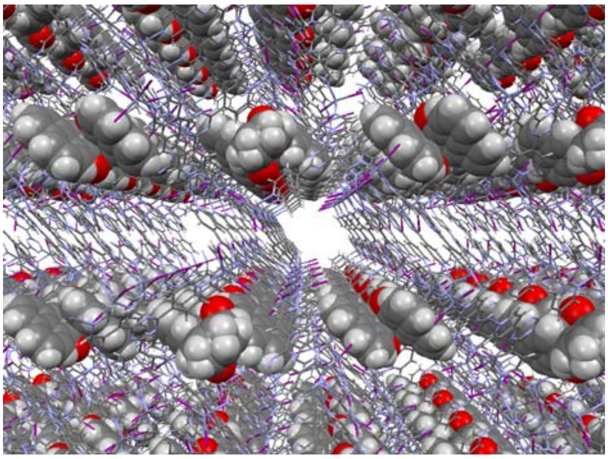

A single crystal X-ray diffractometer (Rigaku XtaLAB P200) equipped with a rotating anode X-ray source (Mo *Kα* radiation) and a hybrid photon counting detector (DECTRIS Pilatus 200K) was used for the diffraction data collection of **6**. The sample crystal was cooled to 93 K using a cold nitrogen stream. Exposure time was 8.0 sec and the number of redundancy was 5.0. The maximum data resolution (*d*) is ca. 0.83 Å. The complete data collection took 9 h. Collected data were integrated, corrected, and scaled using the program CrysAlisPro ver 1.171.38.41. Empirical absorption correction was applied in this process. All crystal structures were solved using the program SHELXT ver. 2014/5 and refined using the program SHELXL ver. 2014/7 as mentioned above.

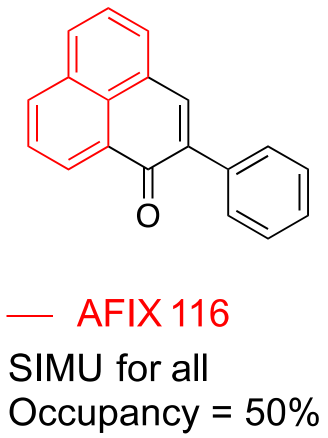

Figure S3. Restrain/constrain chart of compound **3**.

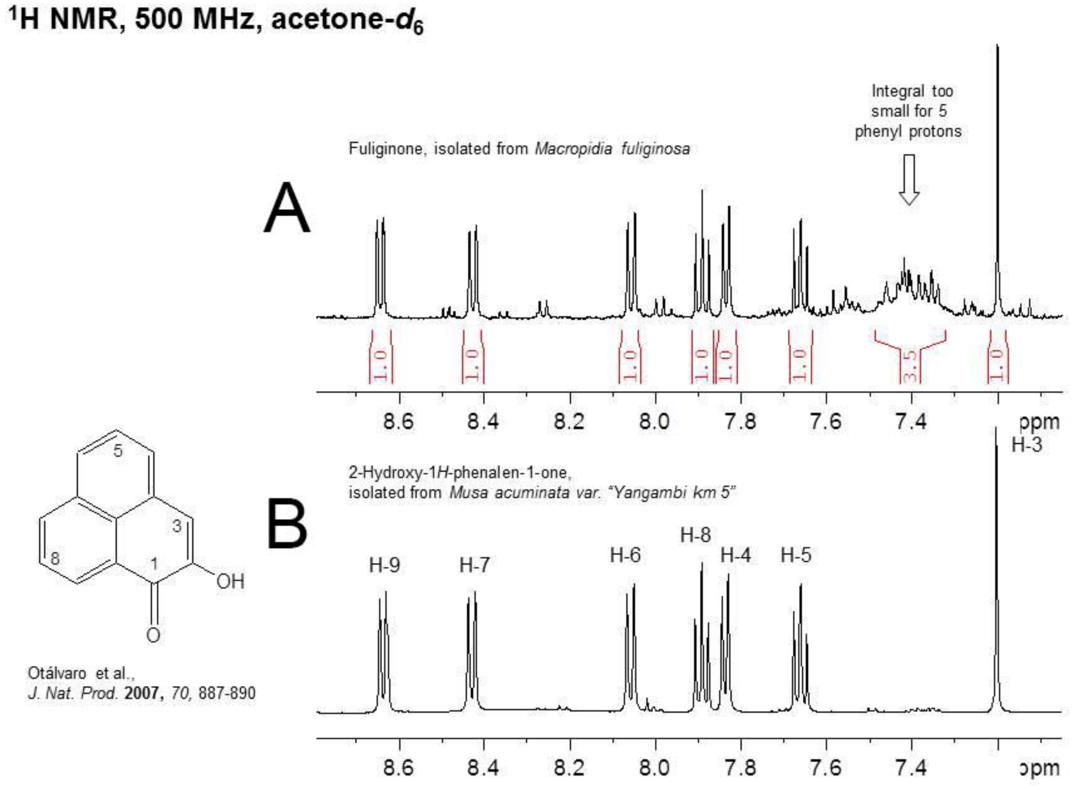

Figure S4. Comparison of the ^{1}H-NMR spectra of the unpurified isolated natural product (**A**) and the synthetic 2-hydroxyphenalenone isolated from *Musa acuminata* var. Yangambi km5.[3] The ^{1}H-NMR spectrum of the isolated compound matches that of synthetic 2-hydroxyphenalenone.

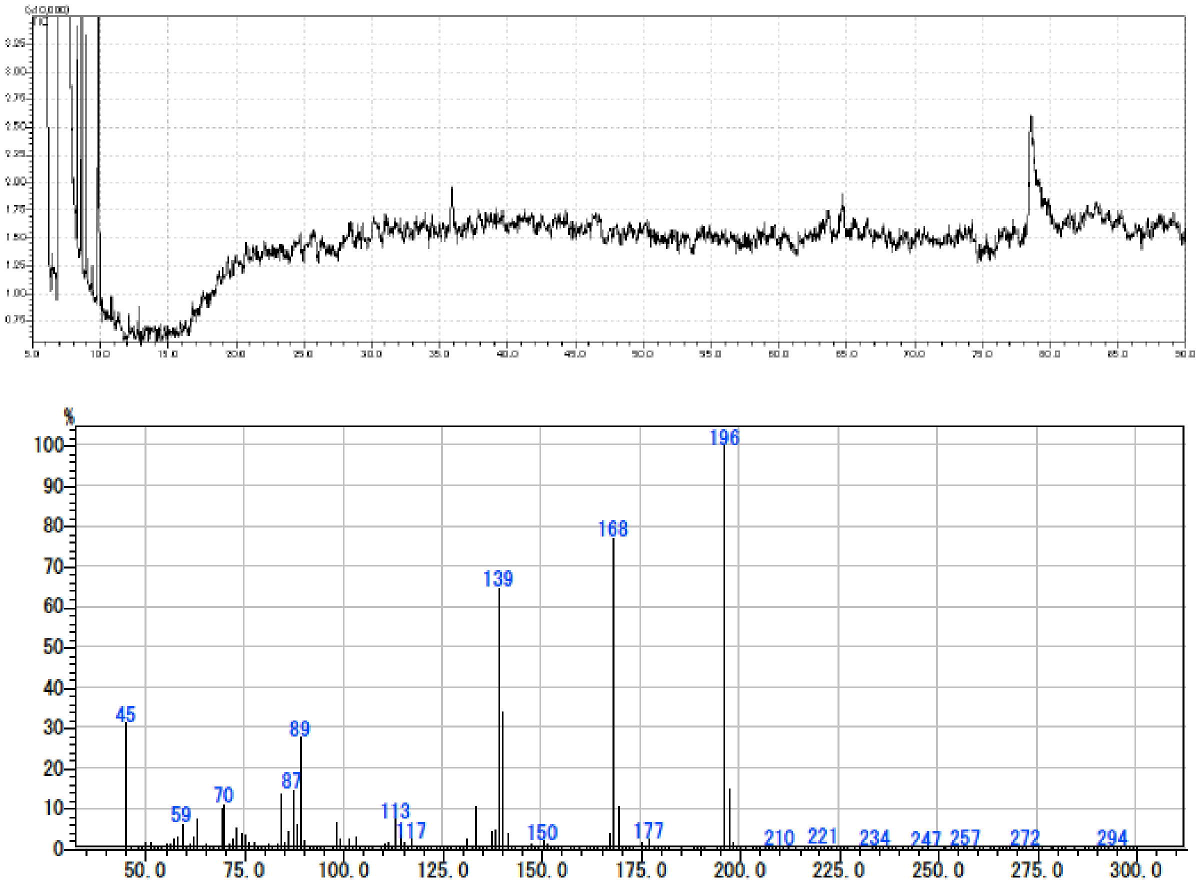

Figure S5. GC/MS chromatogram of the re-purified 2-hydroxyphenalenone.

References

1. Sheldrick, G.M. Crystal structure refinement with shelxl. *Acta Crystallogr., Sect. A* **2015**, *71*, 3-8.
2. Sheldrick, G.M. Crystal structure refinement with shelxl. *Acta Crystallogr., Sect. C* **2015**, *71*, 3-8.
3. Otálvaro, F.; Nanclares, J.; Vasquez, L.E.; Quinones, W.; Echeverri, F.; Arango, R.; Schneider, B. Phenalenone-type compounds from *Musa acuminata* var. "Yangambi km 5″ (AAA) and their activity against *Mycosphaerella fijiensis*. *J. Nat. Prod.* **2007**, *70*, 887–890.
